# LncRNA Gm12664–001 ameliorates nonalcoholic fatty liver through modulating miR-295-5p and CAV1 expression

**DOI:** 10.1186/s12986-020-0430-z

**Published:** 2020-02-04

**Authors:** Qiao Zhang, Jiemei Wang, Hongyin Li, Yuan Zhang, Xia Chu, Jianjun Yang, Ying Li

**Affiliations:** 1 Department of Nutrition and Food Hygiene, Public Health College, Harbin Medical University, Harbin, 150086 China; 2 Department of Public Health College, Kunming Medical University, Kunming, 650550 China; 3 School of Public Health, Ningxia Medical University, Yinchuan, 750004 China

**Keywords:** Nonalcoholic fatty liver, Gm12664–001, miR-295-5p, CAV1

## Abstract

**Background:**

Our study aims to investigate the mechanisms of lncRNA Gm12664–001 improved hepatic lipid accumulation-initiated NAFLD via regulating miR-295-5p and CAV1 in AML12 cells.

**Methods:**

The animals were divided into normal control (NC) group and high fat diet (HFD) group (20 mice per group) for 8w. The steatotic liver was measured by hematoxylin eosin (HE) staining and kits. We performed systematical analyses on hepatic expression profiles of long noncoding RNAs (lncRNAs) and microRNAs in a high-fat diet (HFD)-induced steatotic animal model. The expression profile of targets was confirmed by bioinformatics analysis, luciferase assay, RT-PCR and western blot in AML12 cells.

**Results:**

HFD treatment markedly observed hepatic fatty degeneration with primarily fat vacuoles, and increased TG level compared with control. According to microarray data, we found that transfection of Gm12664–001 siRNA (siRNA-118,306) obviously enhanced TG accumulation and repressed CAV1 in AML12 cells. Furthermore, the TG accumulation markedly increased by siRNA-mediated knockdown of CAV1 in AML12 cells. By bioinformatics prediction, AML12 cells were transfected of siRNA-118,306 obviously upregulated miR-295-5p. Transfection of miR-295-5p mimics significantly increased TG accumulation and obviously suppressed the target CAV1.

**Conclusions:**

The results revealed that lncRNA Gm12664–001 attenuated hepatic lipid accumulation through negatively regulating miR-295-5p and enhancing CAV1 expression in AML12 cells.

## Introduction

Non-alcoholic fatty liver (NAFLD) is a kind of clinical pathological syndrome characterized by excess deposition of fat in liver, which is caused by an imbalance between hepatic synthesis and breakdown of fats, as well as free fatty acids (FFAs) storage and disposal [1]. High fat dietary is closely associated with NAFLD and NAFLD related diseases, including dyslipidemia, body fat deposition, type 2 diabetes mellitus, and thus often regarded as atypical hepatic manifestation of the metabolic syndrome [2]. Furthermore, NAFLD can progress from hepatic steatosis to steatohepatitis, resulting in an increased susceptibility of cirrhosis and hepatocellular carcinoma [3]. Therefore, correcting and decreasing the factors contributing to excessive accumulation of body fat, have been considered effective strategies to improve NAFLD.

Recently, long non-coding RNAs (lncRNAs) are RNA transcripts consisting of more than 200 bp in length and lack protein-coding capacity [4]. Acting as important regulatory molecules, lncRNAs show moderately evolutional conservation and specific transcription and have roles in transcriptional regulation, epigenetic gene regulation, and disease development, such as NAFLD [5]. The report showed that a novel function of lncRNA SRA in promoting hepatic steatosis through repression of ATGL expression [6]. A conserved lncRNA MRAK052686, was found strongly correlated with the antioxidant factor Nrf2, and both genes were down-regulated by the steatotic liver [7].

MicroRNAs (miRNAs) are ∼22 nt non-coding RNA molecules that play important roles in post-transcriptional regulation in plants and animals [8]. Recent research has demonstrated that miRNA are able to regulate adipocyte differentiation, lipid metabolism, and glucose stimulated insulin secretion to exert influence on metabolic pathways that might be also involved in the pathogenesis of NAFLD [9–12]. They have important roles in mammalian development and human diseases such as NAFLD. The study indicated miR-27a is involving in lipid metabolism and inhibits lipid droplets formation in rat hepatic stellate cells [13]. The findings identify miR-132 as a key regulator of hepatic lipid homeostasis [14], miR-29 involved in lipogenic programs of liver [15], and miR-30 regulating hepatic lipoprotein secretion [16]. Also reported as suppressed are miR-29c in diet-induced NASH and miR-21 [17], miR-29c and miR-451 in livers of *ob/ob* mice with fatty liver [18]. In addition, lncRNAs were described to compete for miRNA binding, thereby modulating the derepression of miRNA targets [19].

Therefore, we hypothesized that there might also be certain lncRNA regulating steatosis formation in NAFLD through negatively regulating miRNAs expression and directly targeting downstream molecules. In this study, we performed systematical analyses on hepatic expression profiles of lncRNAs and miRNAs in a high-fat diet (HFD)-induced steatotic animal model. We found out miR-295-5p expression was associated with negatively modulated by lncRNA Gm12664–001, the target gene of Gm12664–001, CAV1, was also the target of miR-295-5p. Then, we further investigated the mechanisms of lncRNA Gm12664–001 could regulate lipid droplets and triglyceride (TG) level in mice hepatocytes by negatively modulating miR-295-5p and directly recognizing and depressing the expression of CAV1, which contributes to the pathogenesis of steatosis formation in NAFLD and might provide a potential novel therapeutic target for the treatment of NAFLD.

## Methods

### Animals

Eight-week-old male C57BL/6 mice (40 mice) were provided by the Vital River Laboratories (Beijing, China) for the experiments. All mice were acclimated for 1 week before initiation of the experiment and maintained on a 12/12 h light/dark cycle with free access to food and water. The animals were divided to the following two groups (20 mice per group) including normal control (NC) group and high fat diet (HFD) group for 8 weeks, the composition of diets in Additional file 1: Table S1. The liver was collected for microarray analysis after 8 weeks of feeding. Experimenters were blind to group assignment and outcome assessment. Animal studies complied with the guidelines of the Harbin Medical University’s Regulations of Animal Experiments and were approved by the Animal Experiment Committee of the Harbin Medical University.

### Microarrays of miRNAs and lncRNAs

The total RNA was extracted from total 6 mice (3 mice per group at 3 replicates) using the TRIzol reagent (Invitrogen, Carlsbad, CA) according to the manufacturer’s protocol. The IncRNAs microarray analysis was performed by KangChen Bio-tech (Shanghai, China). Briefly, the RNA was labeled and hybridized to the Mouse LncRNA Array v2.0 (Arraystar, Rockville, USA), according to Quick Amp Labeling Kit and Gene Expression Hybridization Kit (Agilent Technology, Santa Clara, USA). After washing, the arrays were scanned by the Agilent Microarray Scanner. Agilent Feature Extraction software (version 11.0.1.1) was used to analyze the array images. Quantile normalization and raw data processing were performed using the GeneSpring GX v11.5.1 software package (Agilent Technologies).

### Cell culture, treatment and transfection

Alpha mouse liver 12 (AML12) cells (ATCC, Manassas, VA, USA) were cultured as monolayers in Dulbecco’s modified Eagle’s medium/F12 (GIBCO BRL) supplemented with 10% (v/v) fetal bovine serum (PAA Laboratories, Pasching, Austria). The medium contained HEPES (15 mmol/L), L-glutamine (2.4 mmol/L), pyridoxine hydrochloride (2.4 mmol/L), dexamethasone (40 ng/mL), NaHCO3 (1.2 g/L), penicillin (100 IU/mL) and streptomycin (100 μg/mL), supplemented with ITS (containing 0.005 mg/mL insulin, 0.005 mg/mL transferrin and 5 ng/mL selenium). Cells were grown under an atmosphere of 5% (v/v) CO_2_ in air at 37 °C [20].

Stearic acid and Palmitic acid (Sigma, St Louis, MO, USA) were prepared as previously described [21]. Briefly, stearic acid (SA) or palmitic acid (PA) complex with BSA (3 mM fatty acid: 1.5 mM BSA) was dissolved in ethanol and saponified with sodium hydroxide. After the sodium salt was dried, the sodium salt was re-suspended in saline heated 80 °C until it completely dissolved. When the solution was warm, 20% (w/v) BSA was added and the mixture was stirred at 50 °C for 4 h. Then, the complex was sterilized by filtering for further usage.

AML12 cells were treated with 0, 50, 100, 200, 400 and 800 μM SA or PA for 24 h. Meanwhile, AML12 cells were also exposed to 300 μM SA or 500 μM PA for 24 h. The related indicators were measured after fatty acids treatment.

Small-interfering RNAs (siRNA) against Gm12664–001 was purchased from Santa Cruz Biotechnology (Santa Cruz, CA), and AML12 cells were transfected with siRNA-118306. The scrambled sequence was used as a negative control (NC). MiR-295-5p mimic and miR-183-5p inhibitor were synthesized by RiboBio Co. (Guangzhou, China). The AML12 cells were transfected with 50 nM of miR-295-5p mimic or 100 nM miR-183-5p inhibitor using Lipofectamine2000 (Invitrogen) according to the manufacturer’s instructions.

### Cell viability

After given 0–800 μmol/L SA or PA for 24 h, removing the culture, 200 μL of phosphate buffered saline (PBS) buffer for flushing. Then, 20 μL of MTT solution (5 mg/mL in PBS as the stock solution) was added to the wells, and the plate was incubated for 4 h at 37 °C in the dark. The culture was removed, and 150 μL of DMSO was added to each well and left for 10 min at room temperature. The percentage of viable cells was measured using a plate reader at 570 nm [22].

### Real time-PCR

Total RNAs with miRNAs was isolated with the miRNeasy Mini Kit (Qiagen, Hilden, Germany) according to the manufacturer’s protocol. The miScript SYBR Green PCR Kit (Qiagen) was used to perform qPCR, and the expression level of U6 was used as an internal control. Additionally, all primers were purchased from Qiagen.

### Target prediction and luciferase activity assay

The pmiR-RB-REPORT™ dual luciferase reporter vector carrying the 3′-UTR of miR-295-5p target gene CAV1 was constructed (Ribio Co., Guangzhou, China). AML12 cells were co-transfected with 50 ng of recombinant plasmid, 100 nM of miR-295-5p mimic and mimic NC. After 24 h of transfection, the luciferase activities were measured with a dual luciferase reporter assay kit on a luminometer (GloMaxTM 20/20, Promega).

### Hematoxylin eosin (HE) staining

HE staining was conducted according to routine procedure. In brief, after deparaffinization and rehydration, 5 μm longitudinal sections were stained with hematoxylin solution for 5 min followed by 5 dips in 1% acid ethanol (1% HCl in 70% ethanol) and then rinsed in distilled water. Then the sections were stained with eosin solution for 3 min and followed by dehydration with graded alcohol and clearing in xylene [23].

### TG determination

AML12 cells were transfected with siRNA-118,306, mimic-miR-295-5p, inhibitor-miR-183-5p or siRNA-CAV1 for 48 h. The cells were then washed three times with ice-cold PBS and stained with BODIPY® (Molecular Probes, Eugene, OR, USA) staining solution (diluted with PBS, 1:1000) for 30 min at room temperature. Then cells were then washed again with PBS three times, to remove unbound solution [24]. TG was assayed using commercial kits using enzymatic methods (APPLYGEN, Beijing, China), according to the manufacturers’ instructions.

### Western blot

Western blot was performed as previously described [25] using the following primary antibodies: ATGL, HSL, FAS and CAV1 (Cell Signaling Technology, Danvers, MA, USA) as well as DGAT2 and β-actin (Santa Cruz Biotechnology, Dallas, TX, USA). Anti-rabbit alkaline phosphatase-conjugated antibody (Promega, Madison, WI, USA) was used as a secondary antibody.

### Statistical analysis

Results were presented as mean ± standard deviation. The SPSS 18.0 software was used for all statistical analyses. Statistical analyses were conducted by t test or one-way ANOVA, *P* < 0.05 was considered to indicate a significant difference.

## Results

### High fat diet contributes to lipid accumulation in mice

**Fig. 1:**
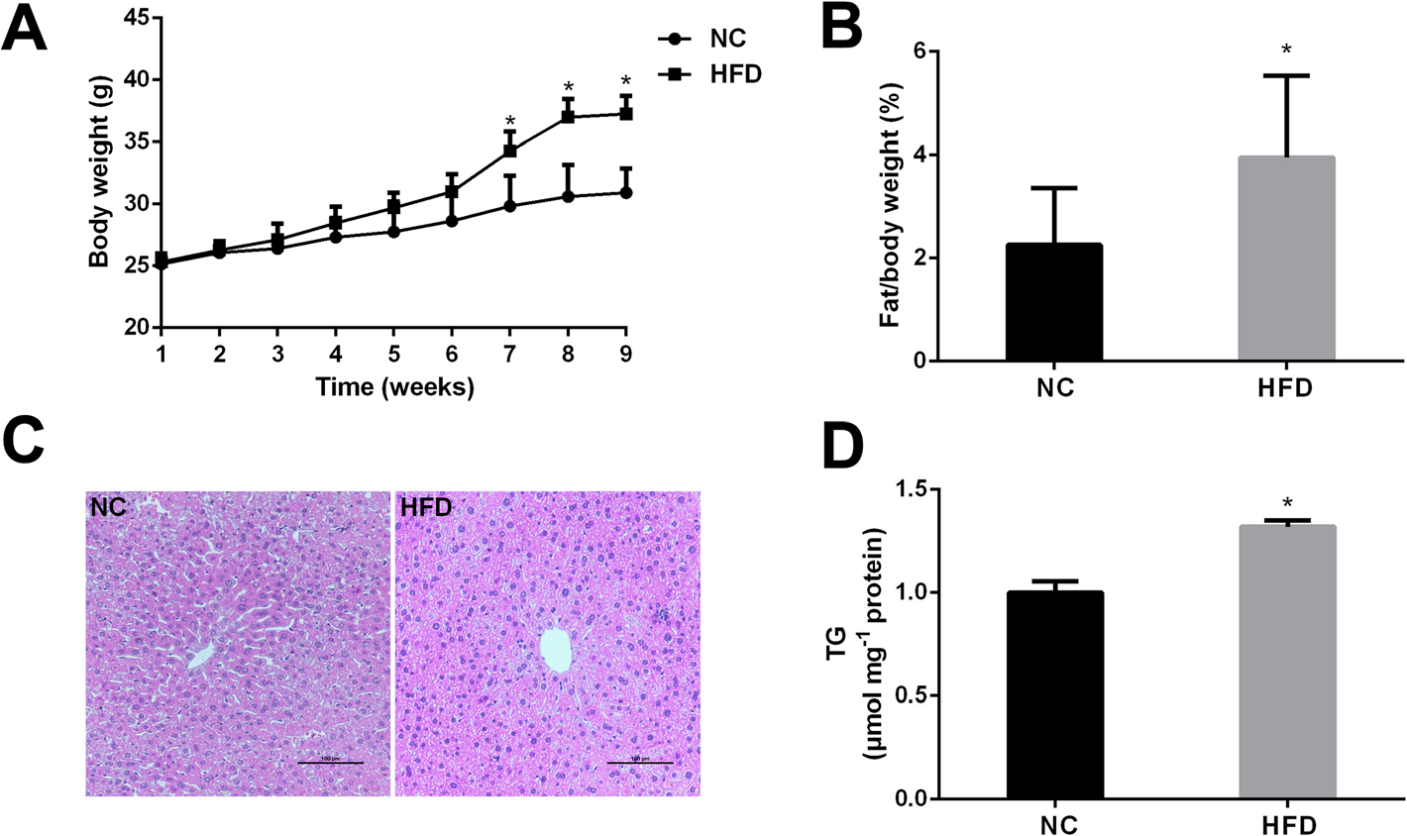
Effects of HFD treatment on lipid accumulation in mice. The body weight (**a**), body fat rate (**b**), HE staining (**c**) and TG level (**d**) after the treatment with HFD in mice. The experiments were repeated 3 times. Data are presented as means ± SD (*n* = 3). * *P* < 0.05, compared with normal control

Compared with normal control, the mice in high fat diet group increased body weight and body fat rate in mice (Fig. 1a, b). The morphological changes of liver were observed by using HE staining (Fig. 1c). HFD treatment markedly observed hepatic fatty degeneration with primarily fat vacuoles was visible, and significantly increased TG level (Fig. 1d), indicating that the high fat diet induced successfully fat mice model with TG accumulation in liver (*P* < 0.05).

### LncRNA Gm12664–001 determination

**Fig. 2:**
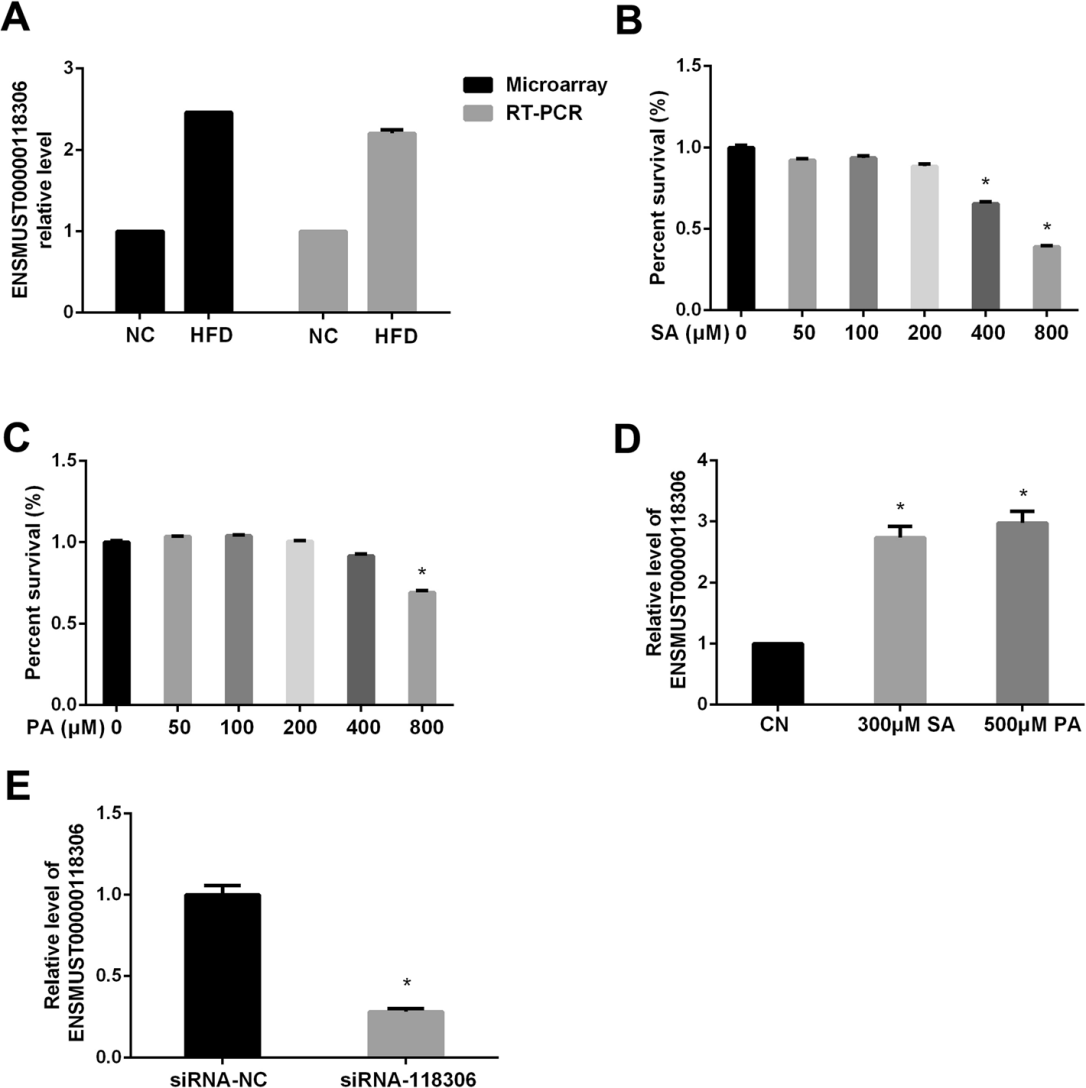
The validation of lncRNAs. The levels of lncRNA Gm12664–001 (ENSMUST00000118306) by microarray and RT-PCR in liver (**a**). The cytotoxicity of 0, 50, 100, 200, 400, 800 μM SA (**b**) or PA (**c**) for 24 h with MTT assay in AML12 cells. The levels of Gm126604–001 after treatment with 300 μM SA or 500 μM PA in AML12 cells (**d**). The level of Gm126604–001 (**e**) after the transfection with siRNA-118,306 in AML12 cells. The experiments were repeated 3 times. Data are presented as means ± SD (n = 3). * *P* < 0.05, compared with control or siRNA-NC

Our microarray data identified 364 upregulated and 387 downregulated lncRNAs, and 29 upregulated and 13 downregulated miRNAs were differentially expressed found in NAFLD samples as compared with the normal liver samples. Among them, lncRNA Gm12664–001 (ENSMUST00000118306) was found to be the most significantly upregulated, and were further verified by RT-PCR (Fig. 2a).

In this study, AML12 cells were treated with 0–800 μM SA or PA for 24 h to evaluate cell viability by MTT assay (Fig. 2b, c). We found that the level of lncRNA Gm12664–001 was markedly increased after treatment with 300 μM SA or 500 μM PA for 24 h in AML12 cells (Fig. 2d). LncRNA Gm12664–001 level was markedly decreased after transfection with Gm12664–001 siRNA (siRNA-118,306) for 48 h in AML12 cells by Real-time PCR (Fig. 2e).

### Effects of lncRNA Gm12664–001 on TG level and the key protein expressions involved in lipid metabolism in AML12 cells

**Fig. 3:**
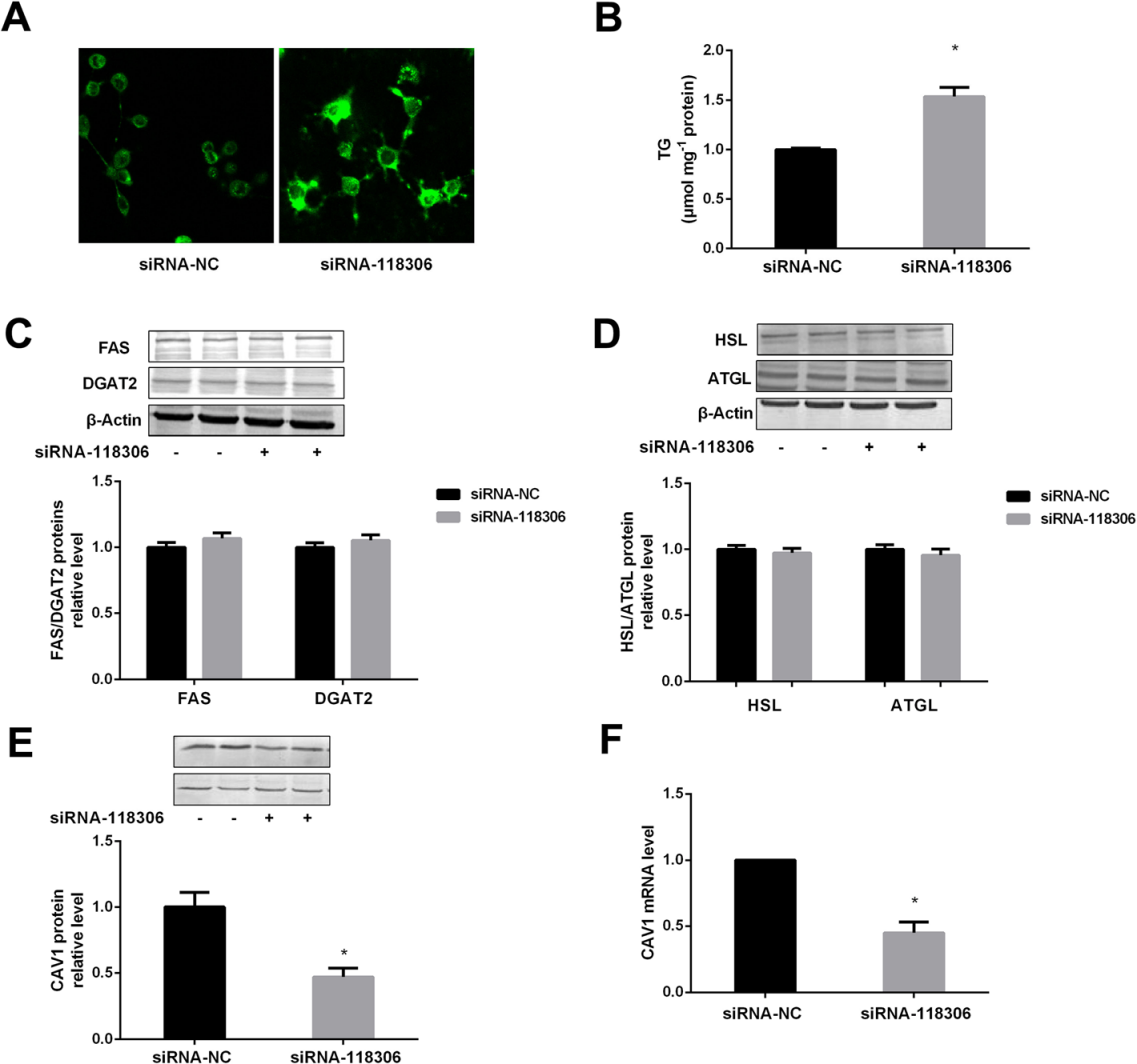
The TG level and protein expressions involved in lipid metabolism in AML12 cells. The lipid droplets (**a**) and TG level (**b**) after transfection of siRNA-118,306 in AML12 cells. The expressions of FAS and DGAT2 (**c**), HSL and ATGL (**d**) after the transfection of siRNA-118,306 for 48 h in AML12 cells. The CAV1 protein expression (**e**) and mRNA level (**f**) after transfection of siRNA-118,306 for 48 h in AML12 cells. The experiments were repeated 3 times. Data are presented as means ± SD (*n* = 3). * *P* < 0.05, compared with siRNA-NC

Lipid droplets and TG level were significantly enhanced after transfection of siRNA-118,306 in AML12 cells (Fig. 3a, b). Moreover, the results suggest that the transfection with siRNA-118,306 has no effect on protein expressions involved the synthesis and decomposition of TG, such as FAS, DGAT2, HSL and ATGL in AML12 cells (Fig. 3c, d). However, CAV1 protein and mRNA levels were mainly involved in transmembrane transport and lipid accumulation markedly suppressed after the transfection of siRNA-118,306 for 48 h in AML12 cells (Fig. 3e, f).

### Effects of CAV1 protein on TG accumulation in AML12 cells

**Fig. 4:**
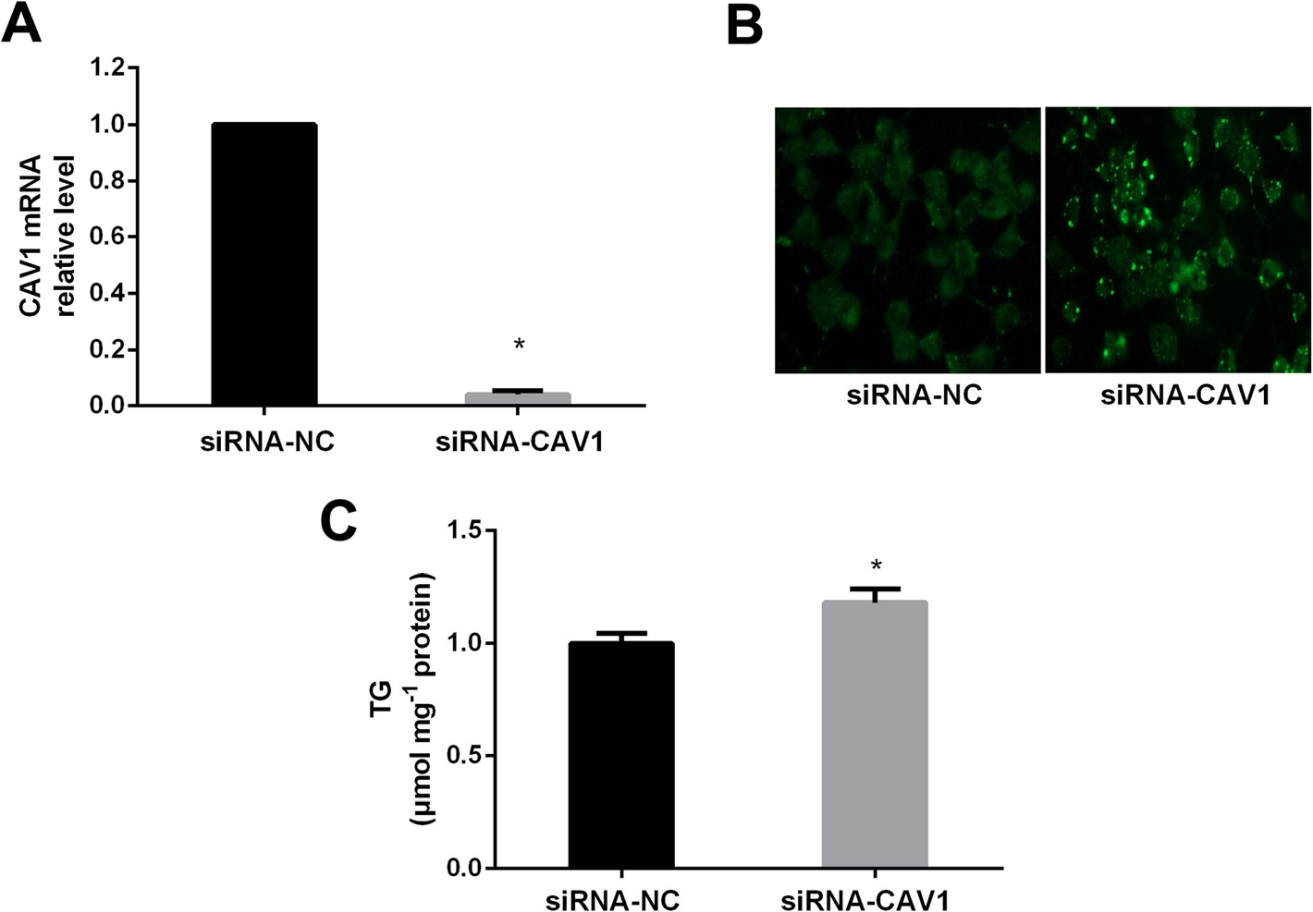
Effects of CAV1 expression on TG accumulation in AML12 cells. The CAV1 mRNA level (**a**), lipid droplets (**b**) and TG level (**c**) after transfection of siRNA-CAV1 in AML12 cells. The experiments were repeated 3 times. Data are presented as means ± SD (*n* = 3). * *P* < 0.05, compared with siRNA-NC

The results suggest that the level of CAV1 mRNA significantly decreased after the transfection of siRNA-CAV1 in AML12 cells (Fig. 4a). The lipid droplets and TG level were obviously increased after transfection of siRNA-CAV1 in AML12 cells (Fig. 4b, c, *P* < 0.05).

### MiR-295-5p contributes to lipid accumulation and CAV1 expression in AML12 cells

**Fig. 5:**
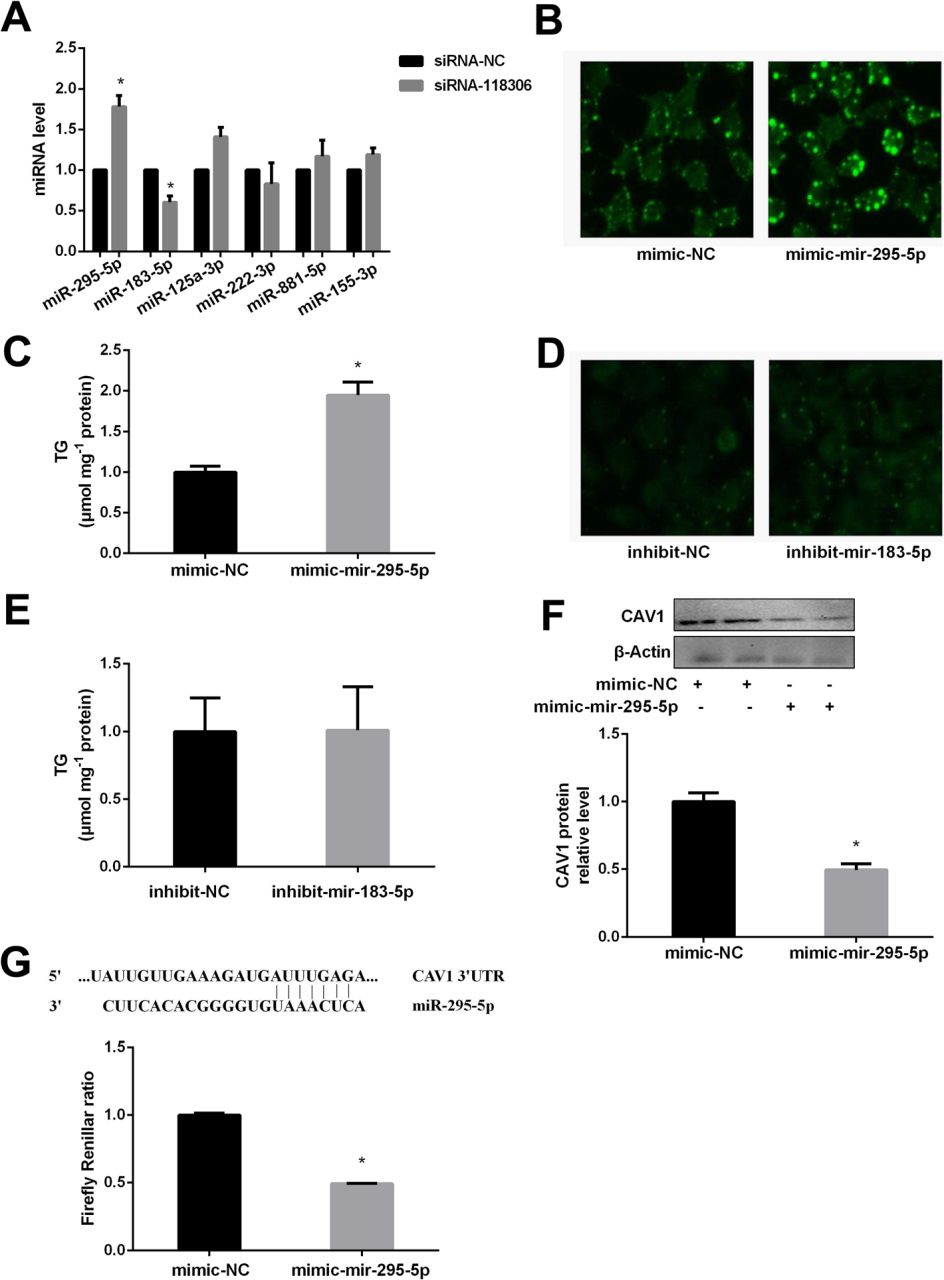
Effects of miR-295-5p on TG accumulation and CAV1 expression in AML12 cells. The expressions of miRNAs (**a**) after the transfection with siRNA-118,306 in AML12 cells. The lipid droplets and TG level after transfection of mimic-miR-295-5p (**b**, **c**) or inhibit-miR-183-5p (**d**, **e**) in AML12 cells. The expression of CAV1 (**f**) and the effects of miR-295-5p on 3′-UTR of CAV1 by luciferase activity assay (**g**) after transfection of miR-295-5p mimic in AML12 cells. The experiments were repeated 3 times. Data are presented as means ± SD (*n* = 3). * *P* < 0.05, compared with mimic-NC

By bioinformatics prediction, we found out 6 miRNAs associated with lncRNA Gm12664–001 among 42 differentially expressed miRNAs. As we expected, AML12 cells were transfected with siRNA-118,306 significantly decreased Gm12664–001 and miR-183-5p levels, and obviously enhanced miR-295-5p expression (Fig. 5a).

Transfection of miR-295-5p mimics significantly enhanced lipid droplets and TG level (Fig. 5b, c), but miR-183-5p inhibitor did not change in AML12 cells (Fig. 5d, e). Moreover, overexpression of miR-295-5p obviously repressed CAV1 protein expression in AML12 cells, and luciferase assay evidently inhibited luciferase activity in AML12 cells further verified that CAV1 was the target of miR-295-5p (Fig. 5f, g).

## Discussion

NAFLD is a kind of chronic liver disease, whose spectrum ranges from simple fatty liver, with benign prognosis, to a potentially progressive form, NASH, which may lead to liver fibrosis and cirrhosis, resulting in increased morbidity and mortality [26]. Without effective medical interventions for complete reverse of NAFLD, it needs to urgently investigate the potential molecular mechanisms of NAFLD to provide a novel the therapeutic strategy for people suffering from NAFLD.

In our studies, we found out HFD induced mice markedly increased body weight, and body fat rate compared with normal control. Additionally, the liver is a major determinant of whole body maintenance of lipid homeostasis and it’s responsible for the synthesis and secretion of fatty acids and TG. Our results suggest that HFD treatment markedly observed hepatic fatty degeneration with primarily fat vacuoles was visible and significantly increased TG level, indicating that the HFD induced successfully high fat mice model with TG accumulation in liver.

LncRNAs are shown to be evolutionally conserved and have roles in transcriptional regulation, epigenetic gene regulation, and disease development, such as embryogenesis, cellular differentiation, tumorigenesis, and hepatic lipid metabolism [27]. LncRNAs were considered to compete for miRNA binding, thereby modulating the expression of miRNA targets. By analyzing gene expression profiles, we identified 751 lncRNAs and 42 miRNAs, which displayed differential expression patterns among the normal control and high fat diet treated mice. We delineated several modules and identified numerous significant genes exhibiting a wide association with liver metabolism and NAFLD-related functions. A significant example of these lncRNAs is Gm12664–001. Our results suggest that lncRNA Gm12664–001 level was markedly up-regulated after 200 μM SA or 300 μM PA-induced TG accumulations in AML12 cells. This result indicated that Gm12664–001 might play a vital role in NAFLD pathological processes. Thus, the outcome might help us provide a new sally port to investigate the underlying molecular mechanisms of NAFLD.

Caveolin-1 (CAV1) is a structural protein of caveolae mainly involved in lipid homeostasis and endocytosis [28]. The reports demonstrate that CAV1 plays an important role in the modulation of lipid metabolism during liver regeneration, and lack of CAV1 alters hepatocyte energy metabolism homeostasis under physiological and pathological conditions [29]. Our results indicated that lipid droplets and TG level were significantly increased after transfection with siRNA-CAV1 in AML12 cells. Additionally, the studies have shown that CAV1 knockout mice display several metabolic abnormalities, including dyslipidemia, hyperglycemia and IR in liver and/or fat tissues [30, 31]. These data further suggest that CAV1 gene was the key molecule of lipid metabolism pathways.

By bioinformatics prediction, we found a strong co-expression of Gm12664–001 and miR-295-5p was demonstrated by either microarray analysis or the experimental data from HFD-fed animals. This implies a potential role of miR-295-5p was negatively modulated by lncRNA Gm12664–001, and significantly decreased lipid accumulation in liver, which associated with CAV1, the gene that are critically involved in NAFLD. Moreover, overexpression of miR-295-5p markedly repressed CAV1 protein expression, and luciferase assay obviously inhibited luciferase activity in AML12 cells further verified that CAV1 was also the target of miR-295-5p.

## Conclusion

The findings of our study clearly showed that lncRNA Gm12664–001 negatively modulating miR-295-5p and repressing the target of CAV1 expression and lowering TG level in liver suggest that lncRNA Gm12664–001 may play important roles in NAFLD by affecting lipid accumulation, which provides possible mechanisms and warrants further investigations.

## Supplementary information

**Additional file 1: Table S1.** The composition of high-fat diet and basic diet.

## Data Availability

All data generated or analysed during this study are included in this published article [and its supplementary information files].

## Abbreviations

AML12: Alpha mouse liver 12
CAV1: Caveolin-1
FFAs: Free fatty acids
HE: Hematoxylin eosin
HFD: High fat diet
LncRNAs: Long non-coding RNAs
MiRNAs: MicroRNAs
NAFLD: Non-alcoholic fatty liver
RER: Respiratory exchange rate
TG: Triglycerides
