# Supplementary material for: LncRNA Gm12664–001 ameliorates nonalcoholic fatty liver through modulating miR-295-5p and CAV1 expression
Source: Nutr Metab (Lond). 2020 Feb 4;17:13. doi: 10.1186/s12986-020-0430-z (PMC7001338; doi:10.1186/s12986-020-0430-z)
Supplement: Supplementary file 1 — Additional file 1: Table S1. The composition of high-fat diet and basic diet. [file 12986_2020_430_MOESM1_ESM.docx]

**Table S1. The composition of high-fat diet and basic diet.**

|  |  | |  | |
| --- | --- | --- | --- | --- |
| Composition | Basic diet (g/kg) | | High-fat diet (g/kg) | |
|  |  |  |  |  |
| Cornstarch | 397. | 486 | 287. | 485 |
| Casein | 200 |  | 200 |  |
| Dextrin | 132 |  | 132 |  |
| Sucrose | 100 |  | 100 |  |
| Lard | 0 |  | 150 |  |
| Soybean oil | 70 |  | 30 |  |
| Cellulose | 50 |  | 50 |  |
| Mixed minerals | 35 |  | 35 |  |
| Mixed vitamins | 10 |  | 10 |  |
| L-Cystine | 3 |  | 3 |  |
| Bile salt | 2. | 5 | 2. | 5 |
| TBHQ (Antioxidant) | 0. | 014 | 0. | 015 |
| Total | 1000 |  | 1000 |  |
| Total energy | 16558. | 86 KJ | 18841. | 34 KJ |
| Fat supply | 15 | % | 36 | % |
